# Comparison of Two Waves of COVID-19 in Critically Ill Patients: A Retrospective Observational Study

**DOI:** 10.1155/2022/3773625

**Published:** 2022-05-31

**Authors:** Kundan R. Jana, Ernie Yap, Kalyana C. Janga, Sheldon Greenberg

**Affiliations:** ^1^Division of Nephrology, Department of Medicine, Maimonides Medical Center, Brooklyn, New York, USA; ^2^Division of Nephrology, Department of Medicine, SUNY Downstate Health Sciences University, Brooklyn, New York, USA

## Abstract

**Background:**

The SARS-CoV-2 virus caused the global COVID-19 pandemic, with waxing and waning course. This study was conducted to compare outcomes in the first two waves, in mechanically ventilated patients.

**Methods:**

This retrospective observational study included all mechanically ventilated COVID-19 patients above 18 years of age, between March 2020 and January 2021. Patients were grouped into first wave from March 2020 to July 2020, and second wave from August 2020 to January 2021. Outcome measures were mortality, the development of acute kidney injury (AKI), and need for renal replacement therapy (RRT). Univariate and multivariate cox regression analysis were used to delineate risk factors for the outcome measures.

**Results:**

A total of 426 patients, 285 in the first wave and 185 in the second wave, were included. The incidence of AKI was significantly lower in the second wave (72% vs. 63%; *p*=0.04). There was no significant difference in mortality (70% vs. 63%; *p*=0.16) and need for RRT (36% vs. 30%; *p*=0.1). Risk factors for mortality were increasing age and AKI in both waves, and chronic kidney disease (CKD) (adj. HR 1.7; 95% CI 1.02–2.68; *p*=0.04) in the second wave. Risk factors for AKI were CKD in both the waves, while it was diabetes (adj. HR 1.4; 95% CI 1.02–1.95; *p*=0.04) and increasing age in the first wave. Remdesivir (adj. HR 0.5; 95% CI 0.3–0.7; *p* < 0.01) decreased the risk of AKI, and convalescent plasma (adj. HR 0.5; 95% CI 0.3–0.9; *p*=0.02) decreased the risk of mortality in the first wave, however, such benefit was not observed in the second wave.

**Conclusions:**

Our study shows a decrease in the incidence of AKI in critically ill patients, however, the reason for this decrease is still unknown. Studies comparing the waves of the pandemic would not only help in understanding disease evolution but also to develop tailored management strategies.

## 1. Introduction

Ever since the identification of the single-stranded ribonucleic acid (RNA) coronavirus, the SARS CoV-2, in January 2020 [1], COVID-19 has emerged as a global pandemic with more than 276 million cases recorded to date and more than 5 million deaths [2]. Of these, 51 million cases and 1 million deaths have been recorded in the USA. Though primarily reported to affect the lungs with interstitial pneumonia worsening to ARDS, COVID-19 has also been reported to be associated with acute kidney injury (AKI), with the virus causing acute tubular necrosis and the viral antigen accumulating in the kidney tubules [3]. Studies so far show the incidence rates of AKI in hospitalized patients to be between 30% and 50% [4–8]. This number was found to be higher in intensive care units (ICU), with incidence rates reaching as high as 78% [9].

The COVID-19 pandemic has been described to occur in “waves” in different countries based on the total number of cases [10–13]. Based on Center for Disease Control (CDC) data, there have been two waves in the USA with a downward national trend during the months of July and August 2020 [14]. Thus, the first wave was between the months of March 2020 to July 2020, and the second wave started from August 2020.

Maimonides Medical Center is a tertiary-level medical center located in South Brooklyn, serving a diverse patient population. It was uniquely positioned to observe the whole spectrum of the pandemic waves as they unfolded and the evolution of standards of care since the discovery of the first case in New York City on February 29, 2020 [14]. In this study, we sought to describe the characteristics of our critically-ill COVID-19 patients between two defined wave time-frames and delineate risk factors for AKI and mortality in them.

## 2. Materials and Methods

This study is a retrospective observational study conducted at Maimonides Medical Center, a 700-bed tertiary-level care hospital in New York City, Brooklyn, USA. All mechanically-ventilated patients above 18 years of age and diagnosed with COVID-19 by reverse transcriptase polymerase chain reaction (RT-PCR) and admitted between March 2020 to January 2021 were included. Patients who were intubated for elective procedures and patients with end stage renal disease were excluded. The respiratory samples were taken from nasal, throat swabs, or endotracheal tube aspirates for diagnostic testing. Institutional Review Board (IRB) approval was obtained to conduct the study (IRB study no. 2020-11-08).

The patients were divided into waves based on CDC data [14]. Patients admitted from March 1, 2020, to July 31, 2020, were grouped under the first wave and those admitted from August 1, 2020, to January 31, 2021, were grouped under the second wave.

Outcome measures were the development of AKI and mortality. AKI was defined according to the KDIGO criteria [15].

Baseline patient characteristics and demographic data were collected, including age, gender, race, body mass index (BMI), past medical history of hypertension (HTN), diabetes mellitus (DM), congestive heart failure (CHF), chronic kidney disease (CKD), and coronary artery disease (CAD). Cerebrovascular disease, CAD, and CHF were combined under cardiovascular disease (CVD). Treatment with remdesivir, dexamethasone, convalescent plasma, and hydroxychloroquine (HCQ) was recorded. In patients who developed AKI, time to the onset of AKI (in days) and need for renal replacement therapy (RRT) were also recorded. Only intermittent hemodialysis and continuous veno-venous hemodialysis were included under RRT. The outcome in terms of death or discharge and length of hospital stay (in days) were also recorded.

Baseline creatinine was established by taking the lowest serum creatinine level in the first week of admission. In patients with elevated creatinine at presentation, we reviewed prior charts within a year to establish the baseline. No ICU protocols for fluid balance were present, and each patient's fluid management was individualized to their volume status.

### 2.1. Statistical Analyses

Continuous variables are presented as mean ± SD. Comparison of continuous variables was performed using one-way ANOVA. Categorical and nominal data were compared using the *χ*^2^ test. Age was entered as a categorical variable (<40, 40 to 70, and older than 70 years). Body mass index (BMI) was entered as a categorial variable (less than 18.5, 18.5 to 24.9, 25 to 29.9, 30 to 34.9, and above 35 kg/m^2^). We used multivariable logistic regression to identify risk factors for AKI and death. The covariates were prespecified on the basis of clinical knowledge and prior studies [6, 16–21]. The univariate and multivariable association of presumed risk factors was performed by the Cox proportional hazards model. All analyses were performed with SPSS, version 26 Armonk, New York.

## 3. Results

A total of 426 patients were enrolled, 241 (56.5%) patients were grouped in the first wave, and 185 (43.5%) patients were grouped in the second wave. Patient characteristics and baseline data are presented in Table 1. BMI was significantly lower in the second wave (30.3 ± 7 vs. 29.3 ± 8.4; *p*=0.045). The proportion of patients with pre-existing cardiovascular disease was significantly higher in the second wave (31% vs. 50%; *p* < 0.01). In comparing COVID-19 directed treatments, there was a significant difference in the utilization of therapeutic agents between the two waves, with the use of HCQ significantly higher in the first wave (79% vs. 1%; *p* < 0.01), and the use of remdesivir (20% vs. 74%; *p* < 0.01), dexamethasone (5% vs. 89%; *p* < 0.01), and convalescent plasma (17% vs. 27%; *p* < 0.01), significantly higher in the second wave.

The incidence of AKI was significantly lower in the second wave (72% vs. 63%; *p*=0.04). There was no significant difference between the two waves in terms of mortality (70% vs. 63%; *p*=0.16) and need for renal replacement therapy (36% vs. 30%; *p*=0.1) (Table 1).

Of the 290 patients with AKI, 97 (33%) required RRT. While CKD increased the risk of requiring RRT (adj. OR 3.02; 95% CI 1.62–5.62; *p* < 0.01), older patients were less likely to undergo RRT (adj. OR 0.97; 95% CI 0.96–0.99; *p* < 0.01). Similar findings were seen in the first wave but not in the second wave (Supplementary Table 1).

Significant risk factors for mortality included increasing age and AKI (adj. HR 2.4; 95% CI 1.7–3.4; *p* < 0.01), while convalescent plasma was associated with decreased mortality (adj. HR 0.7; 95% CI 0.5–0.9; *p*=0.03). While age and AKI were significant risk factors for mortality in both the waves, pre-existing CKD (adj. HR 1.7; 95% CI 1.02–2.68; *p*=0.04) was a significant risk factor for mortality in the second wave. The use of convalescent plasma was significantly associated with lower mortality in the first wave (adj. HR 0.5; 95% CI 0.3–0.9; *p*=0.02), however, it was not observed in the second wave (adj. HR 0.8; 95% CI 0.5–1.3; *p*=0.4) (Table 2).

Mortality analysis based on risk factors, including HTN, DM, Obesity, CKD, and CVD, showed that a composite of 2 or more risk factors was associated with higher mortality overall (HR 1.14; 95% CI 1.04–1.25; *p* < 0.01) and in the first wave (HR 1.28; 95% CI 1.14–1.43; *p* < 0.01), however, such an increased risk was not observed in the second wave. However, after adjusting for age, gender, race, and acute kidney injury, there was no significantly increased risk of mortality among patients with 2 or more risk factors compared to patients with 1 or less risk factors (Table 3).

Risk factors for AKI included increasing age and pre-existing CKD (adj. HR 2.3; 95% CI 1.7–3.2; *p* < 0.01), while remdesivir showed a decreased risk of developing AKI (adj. HR 0.6; 95% CI 0.5–0.8; *p* < 0.01). Pre-existing CKD was found to be a significant risk factor for the development of AKI in both the waves, while increasing age and patients with the history of DM (adj. HR 1.4; 95% CI 1.02–1.95; *p*=0.04) were found to be at an increased risk of developing AKI in the first wave. Remdesivir decreased the risk of AKI in the first wave (adj. HR 0.5; 95% CI 0.3–0.7; *p* < 0.01) but did not have such an effect in the second wave (adj. HR 0.8; 95% CI 0.5–1.4; *p*=0.6) (Table 4).

Among patients who developed AKI, higher age was a significant predictor of mortality, while the use of convalescent plasma decreased the risk (adj. HR 0.7; 95% CI 0.47–0.98; *p*=0.04). Convalescent plasma also decreased mortality in AKI patients in the first wave (adj. HR 0.5; 95% CI 0.29–0.91; *p*=0.02) but similar effect was not seen in the second wave (adj. HR 0.8; 95% CI 0.49–1.29; *p*=0.35) (Supplementary Table 2).

## 4. Discussion

In this single center study of comparing the two waves of COVID-19 patients, we found that there was a significant decrease in the incidence of AKI in the second wave, while the drop in mortality rates and need for RRT did not reach statistical significance. The significant predictors of mortality were increasing age and AKI in both the waves and pre-existing CKD in the second wave. Convalescent plasma was associated with decreased mortality in the first wave. Significant predictors for AKI were increasing age and CKD, and patients with DM in the first wave. Treatment with remdesivir was associated with a decreased risk of AKI in the first wave.

The pathophysiology of AKI in COVID-19 patients is multifactorial. While direct viral infection [22] and overt immune response leading to tubuloepithelial injury and microvascular endothelial injury because of microthrombi formation [23, 24] are some of the underlying pathophysiological mechanisms postulated, autopsy reports showed acute tubular injury as the most common cause of AKI [25]. A few case reports have also described renal infarction in COVID-19 patients because of hypercoagulability [26]. The high incidence of thrombi and intravascular coagulation has been found to be one major difference between COVID-19 and non-COVID-19 AKI [25].

According to a meta-analysis of 142 studies involving 49,048 patients, the pooled worldwide incidence of AKI in hospitalized COVID-19 patients was estimated to be 28.6% [27]. These rates of COVID-AKI have been observed to decrease over time [28, 29]. We found a similar decreased trend in the incidence of AKI in the second wave when compared to the first. One possible explanation could be that the treatment protocols were adjusted to include strategies to prevent AKI during the second wave, having learnt of the potential deleterious effects of SARS-CoV-2 on kidneys, inducing renal failure. Better volume control and different ventilatory strategies could have contributed to the decreased incidence of renal failure [25]. We did not perform a genomic analysis of the virus strain, and hence, the difference in the strain could not be conclusively established as a cause for difference in AKI. We also found no statistically significant difference in mortality and need for renal replacement therapy between the two waves. It could be because of the uniform acuity of our study population, with all of them being critically ill, mechanically-ventilated patients.

The requirement of RRT in COVID-19 patients was previously estimated to be 14% [30], with a varying requirement in the intensive care unit (ICU) setting from 51%–73% [31, 32]. 33% of our patients required RRT (36% in the first wave vs. 30% in the second wave). We found that patients with a history of pre-existing CKD were not only at increased risk for developing AKI but also had increased need for RRT overall, and in the second wave, but not in the first wave. These findings were echoed in previous studies as well [6, 20, 21, 33]. We also found that older patients were less likely to undergo RRT during the first wave but not during the second wave. The discrepancies can be explained by the decision for RRT initiation, which involves complex decision making and judgment, which varies between treating physicians. Apart from this, logistics played a huge role in RRT availability during the first wave when New York was the epicenter for the pandemic [34]. Mortality and severe disease, which preclude the initiation of RRT, could also explain our findings.

An increase in mortality was observed in COVID-19 patients who developed AKI when compared to patients without AKI [6, 20]. Our study is consistent with this, as we observed a two-fold increased risk of mortality with increasing age and development of AKI. In addition, a history of CKD was also associated with 1.5 times risk of mortality in the second wave. While the overall small numbers could explain the discrepancy (13% in the first wave and 18% in the second wave), we also excluded end stage renal disease patients in our study who were described to have high mortality rates, especially during the first wave of the pandemic [35]. Previous studies found the presence of comorbidities like DM and HTN [6, 25] to be the significant predictors of mortality, however, we did not find such an association in our study. One possible explanation is that our study included only mechanically-ventilated patients who were critically ill, whereas other studies included all patients infected with COVID-19. The difference in acuity in study population could be a reason for not finding comorbid conditions as significant mortality risk factors.

While people with DM and older patients were more prone for AKI in the first wave, we could not find a similar increased risk in the second wave. A possible explanation could be that patients in the second wave were comparatively older when compared to the first, although this difference did not reach statistical significance.

The use of remdesivir decreased the risk of developing AKI overall and in the first wave but not in the second wave. We did not find such a benefit with the use of dexamethasone. While there is equivocal evidence on the benefit of remdesivir and dexamethasone in AKI [21, 36], the majority of patients in the second wave (74%) received remdesivir compared to only 20% of patients in the first wave. We also observed that the use of convalescent plasma was associated with improved mortality outcomes in the first wave. Some studies have shown convalescent plasma to have improved outcomes [37, 38], while some others have shown no benefit [39]. There was also a significant difference in the use of dexamethasone (5% in the first wave vs. 89% in the second wave), convalescent plasma (17% in first vs. 27% in second), and hydroxychloroquine (79% in first vs. 1% in second). This discrepancy in the absolute numbers can explain the observed difference in the effects of remdesivir and convalescent plasma on AKI and mortality in the first wave but not in the second wave. Apart from the differences in supportive care, there is also the possibility of a viral mutation, where the kidneys are less frequently targeted.

Our study has the following limitations: it is a single center retrospective analysis, and thus, it is insufficient to draw causality from association. We also do not have information on the respective viral strains. Its strength is that it is a fairly homogeneous patient population, considering the level of acuity being critically ill, requiring mechanical ventilation, and it is drawn from the epicenter of the pandemic across two delineated time points.

## 5. Conclusion

Our study was conducted to understand the difference between the waves of the pandemic, especially in critically ill patients requiring mechanical ventilation. While lessons learnt from the first wave seem to have impacted renal outcomes in the second wave, variants of the virus should also be considered while formulating management strategies. Since the preparation of this manuscript, there have been further waves of COVID-19 with the identification of new viral variants [40]. A continued objective surveillance of clinical outcomes is essential for preparation and improved outcomes in battling this pandemic.

## Figures and Tables

**Table 1:** Comparison of patient characteristics, treatment, and outcomes between the two waves.

Total	First wave	Second wave	*p*-value
	241	185	
Age (years)	66 ± 15.75	70.4 ± 16.5	0.06
Under 40	16	9	
40–70	115	70	
Above 70	110	106	
Body mass index (BMI) (kg/m^2^)	30.3 ± 7	29.3 ± 8.4	0.045
<18.5	3	9	
18.5–24.9	52	56	
25–29.9	76	54	
30–34.9	58	33	
35–39.9	28	15	
≥40	24	18	
African american (AA) race *n* (%)	23 (10)	15 (8)	0.61
Females *n* (%)	83 (34)	79 (43)	0.08

*Past medical history n (%)*			
Diabetes	103 (43)	72 (39)	0.43
Hypertension	160 (66)	127 (69)	0.62
Cardiovascular disease	74 (31)	92 (50)	<0.01
Chronic kidney disease	31 (13)	34 (18)	0.12

*COVID-19 directed treatment n (%)*			
Remdesivir	48 (20)	136 (74)	<0.01
Dexamethasone	13 (5)	165 (89)	<0.01
Hydroxychloroquine	190 (79)	2 (1)	<0.01
Convalescent plasma	41 (17)	50 (27)	<0.01

*Clinical outcomes n (%)*			
Acute kidney injury (AKI)	174 (72)	116 (63)	0.04
Renal replacement therapy (RRT)	62 (36)	35 (30)	0.1
Mortality	168 (70)	117 (63)	0.16

**Table 2:** Comparison of risk factors for mortality.

Covariate	Overall		First wave		Second wave	
	Adj. HR (95% CI)	*p*-value	Adj. HR (95% CI)	*p*-value	Adj. HR (95% CI)	*p*-value
Patient characteristics						
Age						
<40 years	Reference		Reference		Reference	
40–70 years	5.9 (1.85–18.51)	<0.01	8.3 (1.15–59.66)	0.04	2.5 (0.58–10.32)	0.22
>70 years	8.8 (2.79–27.99)	<0.01	10.37 (1.43–75.09)	0.02	5.5 (1.3–23.52)	0.02
African American race	0.7 (0.42–1.16)	0.18	0.7 (0.4–1.31)	0.3	0.7 (0.26–2.06)	0.56
Female sex	1.03 (0.8–1.32)	0.85	0.9 (0.68–1.35)	0.96	1.2 (0.78–1.76)	0.44
Acute kidney injury	2.4 (1.73–3.4)	<0.01	2.1 (1.3–3.27)	<0.01	2.4 (1.42–4.02)	<0.01
Past medical history						
Chronic kidney disease	1.3 (0.95–1.81)	0.1	1.2 (0.76–1.96)	0.41	1.7 (1.02–2.68)	0.12
Diabetes	1 (0.78–1.3)	0.96	1.2 (0.83–1.64)	0.38	0.7 (0.48–1.1)	0.13
Hypertension	0.9 (0.7–1.28)	0.73	1 (0.7–1.56)	0.85	0.8 (0.5–1.26)	0.32
Cardiovascular disease	1 (0.79–1.36)	0.82	1.1 (0.74–1.56)	0.72	1 (0.65–1.56)	0.1
Treatment						
Remdesivir	0.9 (0.61–1.2)	0.38	0.9 (0.54–1.38)	0.54	0.8 (0.47–1.44)	0.5
Dexamethasone	1.1 (0.82–1.57)	0.46	0.2 (0.02–1.04)	0.06	1.6 (0.73–3.67)	0.24
Convalescent plasma	0.7 (0.5–0.96)	0.03	0.6 (0.33–0.91)	0.02	0.8 (0.54–1.28)	0.4

**Table 3:** Mortality analysis with 2 or more risk factors.

	Unadj. HR (95% CI)	*p*-value	Adj. HR (95% CI)	*p*-value
Overall	1.14 (1.04–1.25)	<0.01	0.98 (0.9–1.07)	0.62
First wave	1.28 (1.14–1.43)	<0.01	1.06 (0.95–1.19)	0.3
Second wave	0.99 (0.85–1.15)	0.89	0.88 (0.77–1)	0.06

**Table 4:** Comparison of risk factors for AKI.

Covariate	Overall		First wave		Second wave	
	Adj. HR (95% CI)	*p*-value	Adj. HR (95% CI)	*p*-value	Adj. HR (95% CI)	*p*-value
Patient characteristics						
Age						
<40 years	Reference		Reference		Reference	
40–70 years	2.2 (1.1–4.4)	0.02	2.4 (0.95–6.1)	0.07	1.3 (0.47–3.7)	0.61
>70 years	3.1 (1.54–6.05)	<0.01	2.9 (1.14–7.54)	0.03	2.2 (0.78–6.17)	0.14
Female sex	0.9 (0.71–1.16)	0.43	0.8 (0.59–1.12)	0.21	1 (0.7–1.52)	0.89
Past medical history						
Chronic kidney disease	2.3 (1.67–3.2)	<0.01	2 (1.28–3.2)	<0.01	2.6 (1.62–4.24)	<0.01
Diabetes	1.2 (0.95–1.55)	0.13	1.4 (1.02–1.95)	0.04	1 (0.65–1.41)	0.83
Hypertension	1.2 (0.87–1.54)	0.33	1.1 (0.73–1.61)	0.68	1.2 (0.77–1.85)	0.44
Cardiovascular disease	1.1 (0.85–1.44)	0.44	1.1 (0.78–1.59)	0.55	1.3 (0.83–1.88)	0.29
Treatment						
Remdesivir	0.7 (0.48–0.88)	0.01	0.5 (0.3–0.74)	<0.01	0.9 (0.52–1.43)	0.57
Dexamethasone	0.9 (0.68–1.25)	0.61	0.5 (0.18–1.43)	0.2	1.3 (0.63–2.72)	0.48

## Data Availability

The data used to support the findings of this study are available from the corresponding author upon request.

## Supplementary Materials

Supplementary Materials Supplementary Table 1: the multivariate model for the need for renal replacement therapy (RRT). Supplementary Table 2: comparison of risk factors for mortality in patients developing acute kidney injury (AKI).
