# Supplementary material for: Comparison of Two Waves of COVID-19 in Critically Ill Patients: A Retrospective Observational Study
Source: Int J Nephrol. 2022 May 31;2022:3773625. doi: 10.1155/2022/3773625 (PMC9161135; doi:10.1155/2022/3773625)
Supplement: Supplementary Materials — Supplementary Table 1: the multivariate model for the need for renal replacement therapy (RRT). Supplementary Table 2: comparison of risk factors for mortality in patients developing acute kidney injury (AKI). [file 3773625.f1.docx]

**Supplementary Materials:**

**Supplementary Table 1:** Multivariate model for need for renal replacement therapy (RRT)

| **Covariate** | **Overall** | | **First wave** | | **Second wave** | |
| --- | --- | --- | --- | --- | --- | --- |
|  | OR (95% CI) | p-values | OR (95% CI) | p-values | OR (95% CI) | p-values |
| **Age** | 0.97 (0.96-0.99) | 0.01 | 0.97(0.95-0.99) | 0.02 | 0.97 (0.95-1.01) | 0.1 |
| **Female** | 0.59 (0.34-1.01) | 0.06 | 0.66 (0.33-1.33) | 0.24 | 0.7 (0.28-1.83) | 0.48 |
| **Chronic Kidney Disease** | 3.02 (1.62-5.62) | <0.01 | 1.96 (0.82-4.69) | 0.13 | 7.43(2.48-22.25) | <0.01 |
| **Diabetes** | 1.15 (0.7-1.87) | 0.59 | -- |  | -- |  |
| **Hypertension** | 0.91 (0.53-1.56) | 0.73 | -- |  | -- |  |
| **Cardiovascular Disease** | 1.06 (0.65-1.73) | 0.83 | -- |  | -- |  |

**Supplementary Table 2:** Comparison of risk factors for mortality in patients developing acute kidney injury (AKI).

| **Covariate** | **Overall** | | **First wave** | | **Second wave** | |
| --- | --- | --- | --- | --- | --- | --- |
| **Age** | Adj. HR (95% CI) | p-value | Adj. HR (95% CI) | p-value | Adj. HR (95% CI) | p-value |
| **<40 years** | Reference |  | Reference |  | Reference |  |
| **40-70 years** | 3.2 (1.02-10.36) | 0.05 | 4.3 (0.6-31.4) | 0.15 | 1.3 (0.29-5.91) | 0.73 |
| **>70 years** | 4.4 (1.36-13.95) | 0.02 | 5 (0.69-35.84) | 0.11 | 2.6 (0.57-11.46) | 0.22 |
| **African American race** | 0.6 (0.36-1.12) | 0.12 | 0.7 (0.35-1.28) | 0.23 | 0.6 (0.18-2.01) | 0.41 |
| **Female sex** | 0.9 (0.72-1.26) | 0.72 | 0.9 (0.6-1.27) | 0.49 | 1.1 (0.67-1.79) | 0.66 |
| **Chronic Kidney Disease** | 1.2 (0.86-1.7) | 0.27 | 1 (0.61-1.65) | 0.1 | 1.4(0.83-2.35) | 0.21 |
| **Diabetes** | 0.9 (0.71-1.23) | 0.64 | 0.8 (0.42-1.24) | 0.56 | 0.7 (0.43-1.07) | 0.1 |
| **Hypertension** | 1 (0.71-1.41) | 0.99 | 1.1 (0.72-1.7) | 0.65 | 0.7 (0.43-1.28) | 0.29 |
| **Cardiovascular Disease** | 0.9 (0.7-1.28) | 0.74 | 0.9 (0.67-1.48) | 0.97 | 0.9 (0.51-1.4) | 0.53 |
| **Remdesivir** | 0.8 (0.57-1.21) | 0.33 | 0.7 (0.43-1.27) | 0.28 | 0.9 (0.47-1.6) | 0.65 |
| **Dexamethasone** | 1.2 (0.82-1.67) | 0.43 | -- | 0.95 | 1.6 (0.67-3.95) | 0.29 |
| **Convalescent plasma** | 0.7 (0.47-0.98) | 0.04 | 0.5 (0.29-0.91) | 0.03 | 0.8 (0.49-1.29) | 0.35 |
| **Renal Replacement Therapy** | 0.8 (0.66-1.18) | 0.4 | 0.7 (0.49-1.02) | 0.07 | 1 (0.6-1.72) | 0.97 |
